# Different Non-*Saccharomyces* Yeast Species Stimulate Nutrient Consumption in *S. cerevisiae* Mixed Cultures

**DOI:** 10.3389/fmicb.2017.02121

**Published:** 2017-10-31

**Authors:** Jose A. Curiel, Pilar Morales, Ramon Gonzalez, Jordi Tronchoni

**Affiliations:** Instituto de Ciencias de la Vid y del Vino, Consejo Superior de Investigaciones Científicas – Universidad de La Rioja, Gobierno de La Rioja, Logroño, Spain

**Keywords:** interspecific interaction, biotic stress, non-*Saccharomyces*, mixed starter, wine fermentation

## Abstract

The growing interest of the winemaking industry on the use of non-*Saccharomyces* starters has prompted several studies about the physiological features of this diverse group of microorganisms. The fact that the proposed use of these new starters will almost invariably involve either simultaneous or sequential inoculation with *Saccharomyces cerevisiae* has also driven the attention to the potential biological interactions between different starters during wine fermentation. Our current understanding is that alternative yeast starters will affect wine features by both direct and indirect mechanisms (through metabolic or other types of interactions with *S. cerevisiae*). There are still few studies addressing the question of yeast–yeast interactions in winemaking by a transcriptomic approach. In a previous report, we revealed early responses of *S. cerevisiae* and *Torulaspora delbrueckii* to the presence of each other under anaerobic conditions, mainly the overexpression of genes related with sugar consumption and cell proliferation. We have now studied the response, under aerobic conditions, of *S. cerevisiae* to other two non-*Saccharomyces* species, *Hanseniaspora uvarum* and *Candida sake*, keeping *T. delbrueckii* as a reference; and always focusing on the early stages of the interaction. Results point to some common features of the way *S. cerevisiae* modifies its transcriptome in front of other yeast species, namely activation of glucose and nitrogen metabolism, being the later specific for aerobic conditions.

## Introduction

Employment of non-*Saccharomyces* yeast starters constitutes a growing trend in the winemaking industry. They are proposed as a means to improve aromatic complexity, so recovering some of the features of spontaneous fermentation, while minimizing the risk of microbial spoilage associated to it (Ciani and Comitini, 2011). The potential benefits have been linked to specific yeast species, with commercial strains belonging to *Torulaspora delbrueckii, Pichia kluyveri* or *Lachancea thermotolerans*, among other species. In addition to its contribution to improved aromatic profile, non-*Saccharomyces* strains have been proposed to improve glycerol or mannoprotein content, volatile acidity, color stability, or alcohol level reduction (Ciani and Comitini, 2011; Morales et al., 2015; Ciani et al., 2016).

In terms of microbial interactions, there is a substantial difference between conventional inoculated wine production, in which *Saccharomyces cerevisiae* dominates from almost the beginning of fermentation; and the use of non-*Saccharomyces* starters (either in co-inoculation or sequential inoculation), which results in two different species represented by comparable cell numbers for a relatively long period. Consequently, the contribution of the inoculation of non-*Saccharomyces* strains to winemaking can be either direct or indirect, through biological interactions with *S. cerevisiae*. Some recently described examples include a synergic interaction between *S. cerevisiae* and *T. delbrueckii* resulting in increased levels of 3-sulfanylhexan-1-ol (Renault et al., 2015, 2016) or in a decrease of volatile acidity and higher isoamyl acetate production (Taillandier et al., 2014); synergic interactions between *Debaryomyces vanrijiae* or *Candida sake* and *S. cerevisiae* resulting in enhanced aroma profile (Maturano et al., 2015).

Co-inoculation involving *S. cerevisiae* and other wine yeast species, nearly always results in the disappearance or loss of viability of non-*Saccharomyces* cells (Albergaria et al., 2010; Taillandier et al., 2014; Wang et al., 2015, 2016). Although this dominance can be mainly explained by the indirect impact of sugar consumption rates, nutrient depletion, and ethanol production; some direct mechanisms for yeast species antagonism have also been described. For example, killer factors have been known in *S. cerevisiae* for many years. These secreted peptides, encoded by extrachromosomal elements, affect a limited number of yeast species (van Vuuren and Jacobs, 1992; Pérez et al., 2001). Similar toxins have been described for some other yeast species (Velázquez et al., 2015). In addition, a peptide fragment of the *S. cerevisiae* glycolytic enzyme GAPDH was recently shown to inhibit growth of several wine bacterial and yeast species (Albergaria et al., 2010; Branco et al., 2014).

A few studies have addressed microbial interactions in winemaking by transcriptomic approaches. *S. cerevisiae* was shown to reduce its global transcription activity in co-inoculation with *Hanseniaspora guilliermondii* (Barbosa et al., 2015). In addition, these authors showed that the response of *S. cerevisiae* involved the up-regulation of genes related with biosynthesis of vitamins, and down-regulation of genes involved in the uptake and biosynthesis of amino acids. Rossouw et al. (2012) also identified altered gene expression in *S. cerevisiae* in response to the metabolic activity of *Oenococcus oeni*. The same group identified co-flocculation as a possible mechanism of specific yeast–yeast interspecific interactions (Rossouw et al., 2015). More recently, Pérez-Torrado et al. (2017) analyzed the interaction between different co-inoculated strains of *S. cerevisiae*. The results provided insight on the dominance phenomenon between strains of the same species, highlighting the importance of cell-to-cell contact and differential sulphite production in this process.

The few available genome-wide studies of the interaction between *S. cerevisiae* and non-*Saccharomyces* yeasts (microarray based) analyzed transcription after at least 1 or 3 days of contact between *S. cerevisiae* and the other microorganism. In a recent work, we addressed earlier stages of fermentations co-inoculated with *S. cerevisiae* and *T. delbrueckii*. We found a remarkable transcriptional reprograming for both yeast strains in the presence of each other, as soon as 2 h after being put into contact (Tronchoni et al., 2017). In this work, we have focused on the early transcriptional responses of *S. cerevisiae* to strains belonging to three different yeast species, *T. delbrueckii, Hanseniaspora uvarum*, and *C. sake*. The first species is currently the most widely employed alternative yeast starter for winemaking (e.g., Belda et al., 2015). It was kept as a reference to account for the differences in fermentation conditions between our previous work and the current one (Tronchoni et al., 2017). Species of the genus *Hanseniaspora* are ubiquitous in the winemaking environment, and some of them have been proposed as wine yeast starters (Ciani et al., 2016). *C. sake* has been studied as a promising species for alcohol level reduction in wine by promoting respiratory metabolism (Rodrigues et al., 2016).

## Materials and Methods

### Strains and Media

Four yeast strains have been used in this work, *S. cerevisiae* FX10 (Laffort, SA), a widely used industrial wine yeast strain, *T. delbrueckii* CECT 11199 (CBS 1146), *C. sake* CECT 11909 (CBS 159), and *H. uvarum* CECT 10389 (MCYC 1857). Synthetic must contained (per liter): glucose: 100 g; fructose: 100 g; malic acid: 6 g; citric acid 6 g; YNB w/o aa; w/o (NH_4_)_2_SO_4_ 1.7 g; nitrogen sources (Asp 29 mg; Glu 80 mg; Ser 52 mg; Gln 333 mg; Hys 31 mg; Gly 12 mg; Thr 50 mg; Arg 296.28 mg; Ala 97 mg; Tyr 13 mg; Cys 18.2 mg; Val 29 mg; Met 21 mg; Trp 116 mg; Phe 25 mg; Ile 22 mg; Leu 32 mg; Lys 13.72 mg; Pro 400 mg; NH_4_Cl 306 mg); anaerobic factors (ergosterol 15 mg; oleic acid 5 mg; tween 80 0.5 mL); inositol 18 mg; pH adjusted at 3.5 with NaOH.

### Cultivation Conditions

Pre-cultures were grown in YPD (1% yeast extract, 2% peptone, and 2% glucose) medium for 48 h at 25°C and 150 rpm. Before co-inoculation, pre-cultures were centrifuged at 2200 × *g*, for 15 min at room temperature, and washed twice with distilled water. Mixed cultures constituted by *S. cerevisiae* and one of the assayed strains were inoculated to a total initial optical density (OD_600nm_) of 0.2 (0.1 for each strain) in 200 mL of synthetic must medium contained in 250 mL flasks with wide aluminum foil caps allowing aeration. Single culture of *S. cerevisiae* strain was inoculated to an OD_600nm_ of 0.2 to match conditions in mixed cultures. Flasks were incubated at 25°C under agitation (250 rpm) during 3 h. Experiments were performed in triplicate. The viability of the different populations in the mixed cultures was confirmed after 24 h of co-cultivation. Cells from the mixed cultures were plated at 25°C at different dilutions to ensure individual colony growth and then re-plated at 37°C were *S. cerevisiae* cells can be differentiated from the other yeast species that do not growth at this temperature. This confirmed that after 24 h both yeast species were present in the media.

### RNAseq, Data Analyses, and Statistics

After 3 h of cultivation, total flasks volumes were centrifuged and collected cells washed twice with distilled water before samples were submerged in liquid nitrogen and stored at -80°C for total RNA isolation. Total RNA from the biological triplicates was extracted using RNeasy^®^ mini kit (QIAGEN) and subjected to DNAase treatment using the Ambion DNA-free^TM^ kit according to the manufacturers’ instructions. Concentration, purity, and integrity of RNA samples were determined by spectrophotometric analysis considering the absorbance ratio at 260/280 nm and at 230/260 nm. Library preparation and sequencing of RNA was performed at the Genomics Core Facility in the Center for Biomedical Research of La Rioja (CIBIR). After poly-A filtering, libraries were generated for the different conditions, triplicates of *S. cerevisiae* single cultures and triplicates of *S. cerevisiae* co-cultivated with *T. delbrueckii, C. sake*, and *H. uvarum*. From these libraries, 100-bp pair-end sequence reads were produced with Illumina HiSeq 2000. All raw RNA-Seq data have been deposited in NCBI under Sequence Read Archive SRR5422019 (BioProject PRJNA381847) accession number.

Alignment of reads to the S288c R64 *S. cerevisiae* yeast reference genome assembly was carried out using TopHat2 v.2.0.13 (Kim et al., 2013). Only uniquely mapped single copy, ≤1 polymorphism per 25 bp reads with quality≥ 20 were kept for further analysis. The htseq-count tool (v.0.5.4p5) from HTSeq (Anders et al., 2015) was used to estimate unambiguous read count per genome assembly annotated transcript. Normalization following the trimmed mean of M-values (TMM) method (Robinson and Oshlack, 2010), as well as a time-points DEGs searches (adjusted Benjamini–Hochberg *P* ≤ 0.05 and ≥twofold change) were performed in edgeR v.2.2.6 (Robinson et al., 2010). Finally, fragments per kb of exon per million fragments mapped (FPKM) was calculated using Cuffdiff v.2.2.1 (Trapnell et al., 2013) and low-expressed transcripts were filtered out when FPKM was <1 in both samples. In order to confirm that there was no cross mapping from the co-cultivation, a quimeric genome from *S. cerevisiae* and the non-*Saccharomyces* species was created when the genome was available (*T. delbrueckii* and *H. uvarum*). Almost the same genes (98%) appeared DE comparing both strategies, quimeric and regular mapping.

Different sets of genes were considered for analysis purposes. We refer to significantly up- or down-regulated genes for those that have a log fold change (LogFC) ≥ 0.5 or ≤-0.5 and an adjusted *p*-value ≤ 0.05. When we refer to highly up- or down-regulated genes (a more restrictive category), showing a LogFC ≥ 1 or ≤-1 and an adjusted *p*-value ≤ 0.05. The response of *S. cerevisiae* to each different strain has been analyzed using the more restrictive dataset, including gene ontology analysis. For comparative analysis among the different datasets the broader database has been used (Venn Diagram). Gene expression values showing higher adjusted *p*-values were never taken into consideration for data analysis or discussion, independently of the associated LogFC.

Principal component analysis (PCA) was done using AltAnalyze software (2.1.0) (Emig et al., 2010). AltAnalyze was feeded with the normalized RNAseq data transcripts per million (TPM). The remaining statistical analyses were done using STATA-SE. Venn diagram was done by using Venny 2.1 on-line tool software (Oliveros, 2007–2015). GO term analysis was performed using YeastMine (Balakrishnan et al., 2012). The *p-values* were corrected for multiple testing by the Bonferroni test for functional associations and GO analyses. The statistical level of significance was set at *p*-value ≤ 0.05. Then, GO terms were grouped in biomodules by GO/Module (Yang et al., 2011) to prioritize Gene Ontology.

## Results

### Experimental Set-up

In this work, we have analyzed the effect of three different yeast species over the *S. cerevisiae* transcriptome when grown together at early stages of an aerobic synthetic must fermentation. *T. delbrueckii, C. sake*, and *H. uvarum* were chosen to be co-inoculated with *S. cerevisiae* at equal cell density. After 3 h, cells were sampled and the transcriptome of the *S. cerevisiae* cells from mixed and single cultures was compared by RNAseq analysis. These three yeast species are often isolated from grape must at early stages of fermentation, and are hence natural competitors of *S. cerevisiae* (Fleet, 2003; Jolly et al., 2014). In a previous work the transcriptome of *S. cerevisiae* and *T. delbrueckii* was analyzed after 2 and 12 h of anaerobic co-cultivation. Comparison of single and mixed cultures showed that genes from “Glucose Fermentation Pathway” were overexpressed in both species due to the presence of the other yeast in the media. Overexpression in *S. cerevisiae* is noticed in the first sampling point and in *T. delbrueckii* in the second one. Even though *T. delbrueckii* shows good fermentative fitness in pure culture, it is quickly overtaken by *S. cerevisiae*, perhaps because the earlier reply of *S. cerevisiae*. This made us wonder if the observed quick response of *S. cerevisiae* to the presence of *T. delbrueckii* was specific for this yeast species or similar responses could be obtained with different yeasts. For this reason, other yeast species present at early stages of grape must fermentation but phylogenetically more distant than *T. delbrueckii* (Masneuf-Pomarede et al., 2016) were chosen.

### Genes under NCR Are Induced in the Presence of *T. delbrueckii*

*T. delbrueckii* was used to keep a reference to our published work (Tronchoni et al., 2017) but based on our previous results, the selection of an early time point was set to 3 instead of 2 h. The number of overexpressed genes was similar, with only 44 genes being highly up-regulated (Supplementary File S1). Several genes from the “Glucose Fermentation Pathway” appear significantly up-regulated as well as several genes encoding for glucose transporter proteins, as previously described (Supplementary File S1). Under standard conditions, genes related to fermentation of glucose and its transport into the cell are tightly regulated by the extracellular concentration of glucose through carbon catabolite repression (CCR) (Gancedo, 1998). For instance, high-affinity glucose transporters are only expressed when the concentration of this sugar is low (Ozcan, 2002; Kayikci and Nielsen, 2015). Interestingly, seven out of the eight genes involved in glucose uptake and metabolism are under the control of this carbon source that repress its expression when sugar concentration is high. Based on the glucose concentration of the media, around 200 g/L, these genes should be down-regulated. This result points to a partial relieve of CCR in *S. cerevisiae* by the presence of *T. delbrueckii*.

In addition, Gene Ontology categories enriched for *S. cerevisiae* genes up-regulated in the presence of *T. delbrueckii* were mostly related with nitrogen metabolism, specifically allantoin catabolism (**Table 1**). Actually, most genes induced by co-cultivation with *T. delbrueckii* were involved in utilization of alternative nitrogen sources; and are under nitrogen catabolite repression (NCR) control. The relevance of the activation of these genes is such that five out of the main up-regulated and even half of the highly up-regulated genes were under the control of this transcription factor *GLN3* (Supplementary File S1), required for the expression of genes involved in the use of non-preferred nitrogen sources (Magasanik and Kaiser, 2002). Among them, genes showing the highest over-expression values belong to the *DAL* family. Indeed, the entire pathway for allantoin catabolism was overexpressed.

**Table 1:** Gene Ontology enrichment for *Saccharomyces cerevisiae* in co-cultivation with different non-*Saccharomyces* yeasts.

Co-cultivated yeast species	Regulation	GO IDs	Significance	GO terms
*Torulaspora delbrueckii*	Up	GO:0006144	0.001	Purine nucleobase metabolic process
		GO:0000256	0.000	Allantoin catabolic process
*Candida sake*	Down	GO:0006790	0.002	Sulfur compound metabolic process
		GO:0003333	0.009	Amino acid transmembrane transport
		GO:0046942	0.026	Carboxylic acid transport
		GO:0098656	0.029	Anion transmembrane transport
*Hanseniaspora uvarum*	Down	GO:0009086	0.001	Methionine biosynthetic process
		GO:0019379	0.000	Sulfate assimilation, phosphoadenylyl sulfate reduction
		GO:0070814	0.004	Hydrogen sulfide biosynthetic process

Like the CCR and NCR dependent genes mentioned above, expression of other genes expected to show low activity in rich medium, especially after only 3 h of incubation (i.e., before actual consumption of carbon or nitrogen sources might be observed), was also highly induced in *S. cerevisiae* by co-cultivation with *T. delbrueckii*. Among them, we found other NCR dependent genes like those coding for proline permease, *PUT4*; proline oxidase, *PUT1*; general amino acid permease, *GAP1*; GABA permease, *UGA4*; or a putative allantoate permease; as well as some high affinity permeases and metal transporters like those for inorganic phosphate, *PHO84*; sulfate, *SUL1*; copper, *CTR3*; or cysteine, *YCT1* (Supplementary File S1).

### Genes Involved in Cell Replication Are Up-regulated by Co-cultivation with *C. sake*

No significant gene ontology enrichment was found for the only 20 genes showing a high overexpression in *S. cerevisiae* when co-cultivated with *C. sake*. About half of them were shared with the list of highly overexpressed genes coming from *T. delbrueckii* co-cultivation, including genes already discussed above, related with the allantoin pathway, nitrogen uptake or non-preferred nitrogen sources, and genes involved in glucose uptake and metabolism (Supplementary File S1). Some of these genes in common showed higher expression compared to the *T. delbrueckii* experiment, like *CHA1*, involved in the use of nitrogen sources (serine or threonine). Other highly overexpressed genes shared between *C. sake* and *T. delbrueckii* co-cultivation are all involved in replication (specially RNA helicases, but also rRNA and ribosome biogenesis, or Start checkpoint), or related with cell wall (*TIP1*), and membrane lipid composition (*OLE1, ERG5, ERG3, ERG11, ERG1*, and *ERG25*) (Supplementary File S1). The up-regulation of these genes suggests another possible strategy of *S. cerevisiae* to improve competitiveness in grape must, besides or complementary to the activation of genes required for sugar and nitrogen consumption. This would consist of an increase in relative membrane surface (through increased cell numbers), which will help accelerate nutrient uptake in detriment of other yeasts.

In contrast to the *T. delbrueckii* experiment, in *C. sake* competition, there are more genes highly down-regulated than up-regulated (34 genes). These genes are summarized in the GO term categories “carboxylic acid transmembrane transport” and “sulfur compound metabolic process” (Supplementary File S1). Some of the genes under these two categories are *MET1, MET2, MET8, MET32, ISU2*, or *SUL2*. They encode methionine and sulfur permeases, and are involved in methionine synthesis, or the synthesis of iron-sulfur proteins. Thus, the transcriptional response of *S. cerevisiae* to co-cultivation with *T. delbrueckii* or *C. sake* is similar, considering overexpressed genes. However, there are clear differences among the down-regulated genes. Actually, from the 34 genes highly down-regulated in the *C. sake* experiment, only four appear in the *T. delbrueckii* down-regulated dataset, while other two genes appear as up-regulated (Supplementary File S1).

### Co-cultivation with *H. uvarum* Triggers the Expression of Genes under “Response to Stress” Category

Co-cultivation with *H. uvarum* resulted in a low gene expression profile as seen with *T. delbrueckii* and *C. sake*, with 29 genes showing high overexpression, 6 genes in common with *T. delbrueckii*, and only 3 with *C. sake* (Supplementary File S1). Among the 29 genes significantly highly up-regulated in these cultures 12 out of them belong to the GO categories “response to stimulus” and/or “response to stress.” Thus, many of the most overexpressed genes are involved in resistance to several stresses, for instance, pleiotropic drug resistance (2 genes), heat (4 genes), DNA replication stress or DNA damage (6 genes) or osmotic stress (Hog1 dependent, 2 genes). It is also worth mentioning three genes coding for cell wall mannoproteins (*TIR1, TIP1*, and *DAN1*), among the 10 most overexpressed genes. These mannoproteins belong to the Srp1/Tip1 family and have been described to respond to different stresses like cold stress and to be stimulated in the adaptation to hypoxia (Sertil et al., 1997; ter Linde et al., 1999; Abramova N. et al., 2001; Abramova N.E. et al., 2001; Tai et al., 2005). The results obtained for *H. uvarum* co-cultivation suggest that some cell wall proteins might be also important for the adaptation of *S. cerevisiae* to biotic stress.

As seen with *C. sake* mixed cultures, transcriptional response to co-cultivation with *H. uvarum* results in the repression of a great number of genes in *S. cerevisiae*, 31 in this case highly down-regulated (Supplementary File S1). This response is similar to that observed for *C. sake*, with several genes showing a reverse behavior, as compared to the *T. delbrueckii* experiment (7 genes). Some of these genes were important in the discussion of the effect of *T. delbrueckii* over *S. cerevisiae* in mixed cultures made above (*HSP12* and *PDC5*) and in our previous work (Tronchoni et al., 2017), like *HSP12* (described by us and others in *S. cerevisiae* – *S. cerevisiae* interactions).

### Different Yeast Species Promote a Different Response in *S. cerevisiae* Although Some Similarities Can Be Observed

**FIGURE 1:**
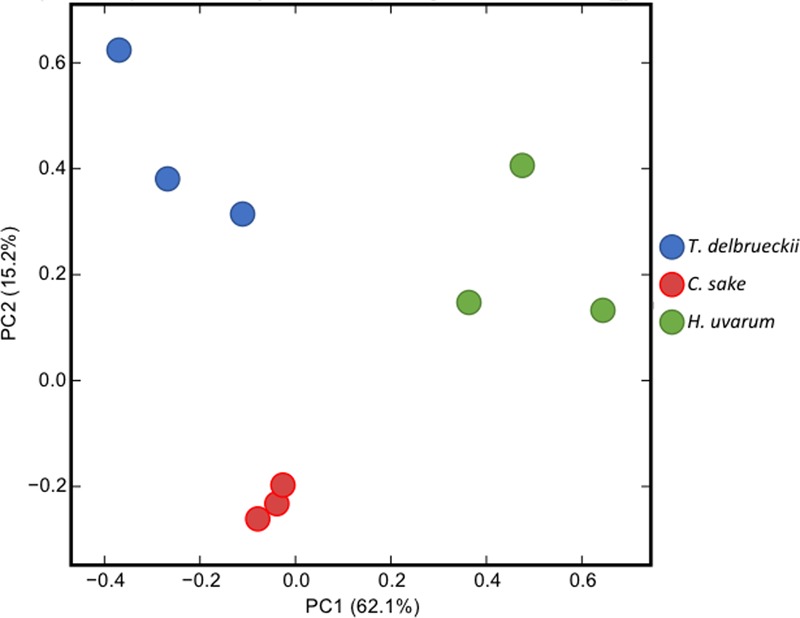
Principal component analysis (PCA) of the normalized RNAseq data transcripts per million (TPM) of *Saccharomyces cerevisiae* in response to co-cultivation with different yeasts species.

Total LogFC datasets without threshold restrictions were used to perform a PCA to compare the responses of *S. cerevisiae* to the different yeasts in co-cultivation (**Figure 1**). Results cluster each independent replicate together for each yeast, although there is a higher dispersion of *T. delbrueckii* and *H. uvarum* compared with *C. sake*. Principal Component 1 (PC1) explaining 62.1% of the variance separates *T. delbrueckii* and *C. sake* from *H. uvarum*, and PC2 explaining 15.2% of the variance, *T. delbrueckii* and *H. uvarum* from *C. sake*. Thus, each yeast ends up in a different section of the PCA. Highlighting a particular response of *S. cerevisiae* when co-cultivated with different yeast species.

**FIGURE 2:**
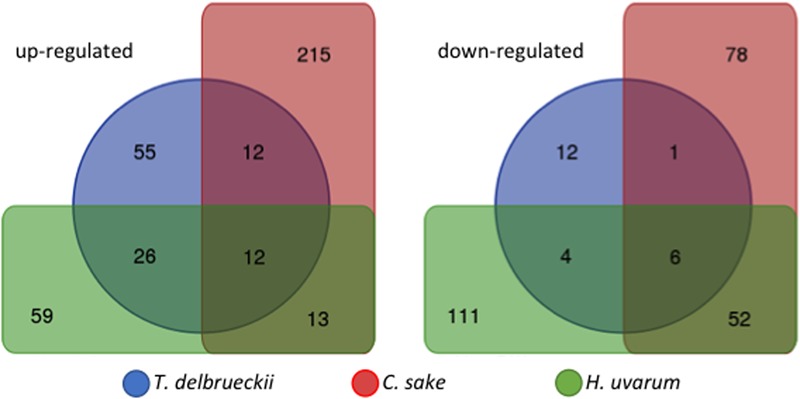
Venn diagram showing up-regulated and down-regulated significant genes (LogFC ≥ 0.5 or ≤-0.5; *p*-adjusted ≤ 0.05) of *S. cerevisiae* in response to co-cultivation with different yeasts species.

Although PCA results define a different response depending on the yeast mixed culture, the co-cultivation experiments shared global transcriptomic characteristics as well as genes behaving in a similar manner. One of the main characteristics shared by all the experiments carried out so far is that it is a moderate response, gene expression fold changes are low as well as the number of significant genes. Venn diagram showing significant up-regulated genes (LogFC ≥ 0.5; *p*-adjusted ≤ 0.05) for the three species tested shows the degree of similarity described (**Figure 2**). Although there are genes in common among them, the percentage varies from the 35% of the genes being in common between *T. delbrueckii* and *H. uvarum*, both with similar number of up-regulated genes to the much lower number of genes shared with *C. sake* or among the three of them. On the other hand, Venn diagram for down-regulated genes (LogFC ≤-0.5; *p*-adjusted ≤ 0.05) shows a much more heterogeneous response to co-cultivation depending on the competing yeast species.

**FIGURE 3:**
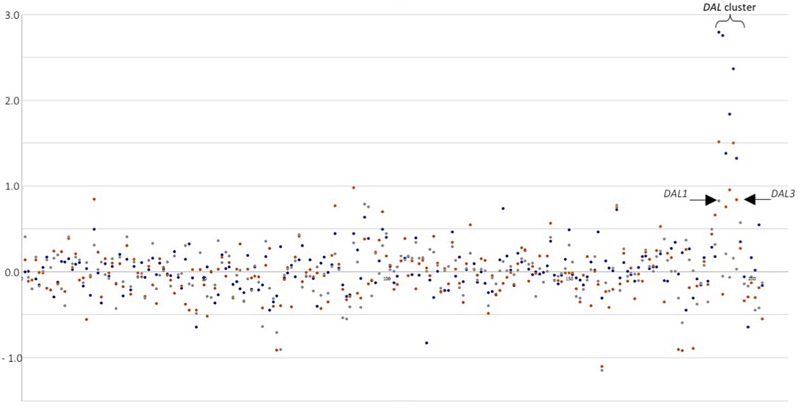
Differential expression (LogFC) for genes in chromosome IX of *S. cerevisiae* in response to co-cultivation with different yeasts species. *DAL* cluster is highlighted. Arrows point first and last gene in the cluster. Blue dots: *T. delbrueckii* mixed culture. Orange dots: *C. sake* mixed cultures. Gray dots: *H. uvarum* mixed culture.

A general picture can be drawn from the short list of genes equally up- or down-regulated in *S. cerevisiae* in response to co-cultivation among the three experiments (**Figure 2**). From the 12 genes commonly up-regulated, 3 are involved in glucose uptake and glycolysis, and according to literature should be repressed by high levels of glucose (after 3 h of co-cultivation in synthetic must, glucose concentration is close to 200 g/L). *HXT12*, a high-affinity glucose transporter and both cytoplasmic and mitochondrial aldehyde dehydrogenases; other three involved in membrane lipid metabolism, *OLE1* (monounsaturated fatty acid synthesis), *FAA4* (long-chain fatty acyl-CoA synthetase), and *ERG5* (ergosterol biosynthesis pathway); also, the major cell wall mannoprotein *TIP1*; two more related with the nitrogen sources available, *CHA1* (serine or threonine) and *PUT1* (proline). The three remaining genes codified for a protein required for antifungal drug resistance (*COS111*), a membrane protein involved in zinc ion homeostasis (*IZH1*) and *INA1* a putative protein of unknown function which paralog is *FAT3*, a protein required for fatty acid uptake. Therefore, as has been described previously for each individual yeast co-cultivation, this set of genes can be summarized in glucose uptake, membrane and cell wall biogenesis, and nitrogen utilization. Thus, despite the clean separation of the three yeast species co-cultivated by the PCA there are some trends common to all experiments. This can be seen in the expression of the *DAL* family of metabolic genes. Plotting LogFC for the different co-cultivation vs. single *S. cerevisiae* cultures of chromosome IX reveals the induction of this gene cluster (**Figure 3**), showing higher overexpression values for *T. delbrueckii*, lower values for *C. sake* despite a clear trend is observed, and just one gene significative for *H. uvarum*.

## Discussion

In a previous article, we analyzed the transcriptional response to co-cultivation of *S. cerevisiae* and *T. delbrueckii*. The study focused in the initial stages of wine fermentation, before *S. cerevisiae* completely dominated the mixed cultures. Both species showed a clear response to the presence of each other, even though the portion of the genome showing altered transcriptional levels was relatively small. Changes in the transcription pattern suggested a stimulation of metabolic activity and growth. Specifically, gene expression of the glucose fermentation pathway was induced. This was observed for both yeast species. However, the timing was different, with *T. delbrueckii* showing a delayed response (12 h) as compared to *S. cerevisiae*.

The early response of *S. cerevisiae* after 2 h of co-cultivation decided us to focus at this first time point in this new work. The selection of an early time point ensures that the gene expression changes are responding to the direct presence of the other yeast species instead of other more indirect signals like faster nutrient depletion from the media. In order to allow higher transcriptomic changes compared to previous results, cells were collected at 3 h of co-cultivation. Here we addressed the species-specificity of this early response to biotic stress, by co-cultivating *S. cerevisiae* with phylogenetically more distant yeast species. Three different species, common in the wine fermentation environment, were selected, *T. delbrueckii*, in order to have a reference to previous experiments, C. *sake*, and *H. uvarum* (Masneuf-Pomarede et al., 2016). *H*. *uvarum* was chosen because is one of the most abundant yeast species found on grapes and in grape must (Albertin et al., 2015), therefore usually present when *S. cerevisiae* is inoculated, but also because it has been proposed as a non-*Saccharomyces* starter (Tristezza et al., 2016). *C*. *sake* has also been proposed to be co-inoculated with *S. cerevisiae* in order to improve and differentiate the wine fermentation process (Maturano et al., 2015; Rodrigues et al., 2016). This experiment was carried out under aerobic regime to better understand the behavior of yeast under this condition, that has been proposed as an alternative to reduce alcohol in wines by using non-*Saccharomyces* species in co-cultivation. In this occasion, the stimulation of metabolic activity previously seen for *S. cerevisiae* in co-cultivation with *T. delbrueckii* was confirmed in this work, not only for glucose metabolism but also for nitrogen metabolism. Several of the induced genes are described as being under NCR control. Apparently under conditions of co-cultivation with *T. delbrueckii, S. cerevisiae* partially relieves the nitrogen and glucose catabolite repression, up-regulating a series of genes that, in pure culture, are usually expressed in later stages of growth in grape must, when the concentration of easily assimilated carbon and nitrogen sources has decreased. An explanation for this could be that *S. cerevisiae* is responding by increasing the flux of nutrients (glucose and nitrogen) to reduce their availability for *T. delbrueckii*. A common response observed in both species in the different time points (2 and 12 h) in our previous work showed *HSP12* as a possible marker for co-cultivation. Current results confirm its induction after 3 h, but do not support the view of *HSP12* induction as a general response to co-cultivation in *S. cerevisiae*, since its expression was down-regulated in front of the other yeast species. However, overexpression of *HSP12* might depend on the competition strength of the strain in co-cultivation, or on the nature of the relationships established between the two strains in the mixed culture (cooperative or antagonistic). Therefore, up-regulation of *HSP12* will only take place under conditions of co-cultivation that may pose a challenge to the growth of *S. cerevisiae* cells.

The effect of the other two phylogenetically more distant yeasts, *C. sake* and *H. uvarum*, over *S. cerevisiae* gene expression was also examined after 3 h of co-cultivation under aerobic conditions. A set of genes related to glucose and nitrogen metabolism as observed in *T. delbrueckii*, appeared also overexpressed in the mixed culture, as compared to *S. cerevisiae* single cultures, although it does not involve as many genes as in the case of *T. delbrueckii*, and the overexpression levels are also lower. On the other hand, among the genes up-regulated there were several not observed for *T. delbrueckii*. The specific set of genes responding to co-cultivation with *C. sake* play different functions, including cell replication (genes involved in ribosome biogenesis, RNA helicases or Start checkpoint) or genes related to membrane maintenance. This could be pointing to a second strategy, compatible and complementary to the metabolic stimulation. The increase in population size by accelerating cell division. This would help increase the uptake of nutrients, decreasing their availability for competitor yeasts. Co-cultivation with *H. uvarum*, also induced genes that point to cell duplication as a target to improve competitiveness by *S. cerevisiae*.

Overexpression of the DAL family of genes has revealed in this work as a diagnostic feature of the relief of nitrogen catabolite repression in response to co-cultivation under aerobic conditions. Allantoin metabolic and catabolic processes appear as significantly enriched GO terms in response to *T. delbrueckii* (**Table 1**). And, although not statistically significant, the same trend was observed in the response of *S. cerevisiae* to the other two yeast species, as illustrated in **Figure 3**. The weaker impact on DAL expression levels in *C. sake* or *H. uvarum* co-cultures, as compared to *T. delbrueckii* might be related to the closer phylogenetic proximity of the later with *S. cerevisiae*, or the nature of the established interactions (positive or negative). This pathway has been described before to be relevant in yeast–yeast interactions. In comparisons of *S. cerevisiae* single cultures vs. mixed cultures with a non-*Saccharomyces* species (*H. guilliermondii*), the allantoin pathway was significant in single cultures after 24 h of cultivation (Barbosa et al., 2015). In our previous work that matches better their experimental conditions under anaerobiosis, after 2 h of co-cultivation, single cultures of *S. cerevisiae* had some genes from the allantoin pathway significantly up-regulated, but not enough genes to have the GO-term significantly expressed. On the contrary, in this work, it is the co-cultivation after 3 h what triggers this pathway in *S. cerevisiae*. Since the DAL gene cluster is up-regulated this time as well as many genes related to nitrogen metabolism and the uptake of non-preferred nitrogen sources, these differences should be due to the aeration regime selected under co-cultivation conditions in each experiment. It is concluded that the culture conditions have a strong impact on the way *S. cerevisiae* responds to the presence of competing yeast species. Indeed, overexpression of NCR dependent genes, including DAL genes, was not appreciated in experiments performed under anaerobic conditions (Tronchoni et al., 2017). Probably this is related to the stimulation of biomass production due to oxygen availability and partial respiratory metabolism. Further stimulation due to co-cultivation would hence lead to an increased demand of nitrogen, and the consequent overexpression of genes required for the assimilation of alternative nitrogen sources.

*S. cerevisiae* and *T. delbrueckii* responses to each other were similar in our previous work. They also showed similarities with other yeast–yeast interactions between *S. cerevisiae* strains (Rivero et al., 2015). This work confirms our previous results with *T. delbrueckii*, but also that, despite some similarities, the differences are enough to distinguish the effect of each yeast species. Interestingly there are also examples, like the DAL family of genes, were the same genes are involved in the responses of all species, but with clear differences in the intensity of the response. Therefore, although co-culture with different yeasts produces a similar response, this is not the exact same, and at least with the yeasts analyzed in this work each one induces a particular profile of gene expression in *S. cerevisiae*.

This work confirmed metabolic stimulation in *S. cerevisiae* as a consequence of co-cultivation with different wine yeast species, in synthetic must. This response was stronger for *T. delbrueckii*, which is a close phylogenetic relative of *S. cerevisiae*, than for not so closely related species. This response involves, by one side, overexpression of genes in the gluco-fermentative pathway; and by the other side, a partial relief of NCR. The later seems to depend on oxygen availability. In addition, the response to *C. sake* and *H. uvarum* suggests a complementary strategy, enhancing cell duplication rates. Our results contribute to better understanding the behavior of starter yeasts in co-culture (*S. cerevisiae* with non-*Saccharomyces* strains), a promising winemaking practice whose application is steadily increasing in the cellars.

## Availability of Data and Material

The data set supporting the results of this article is available in the NCBI repository under Sequence Read Archive SRR5422019 (BioProject PRJNA381847) accession number (http://www.ncbi.nlm.nih.gov/Traces/sra/sra.cgi?view=announcement). The data set supporting the results of this article is included in the article (and its Additional files).

## Author Contributions

JT, RG, PM, and JC conceived and designed the study. JC performed the experiments. JT and JC, analyzed the data. JT, RG, and PM interpreted the results and wrote the manuscript. All authors discussed and approved the manuscript.

## Conflict of Interest Statement

The authors declare that the research was conducted in the absence of any commercial or financial relationships that could be construed as a potential conflict of interest.

## References

[B1] Abramova N. Sertil O. Mehta S. Lowry C. V. (2001). Reciprocal regulation of anaerobic and aerobic cell wall mannoprotein gene expression in *Saccharomyces cerevisiae*. *J. Bacteriol.* 183 2881–2887. 10.1128/JB.183.9.2881-2887.2001 11292809 PMC99506

[B2] Abramova N. E. Cohen B. D. Sertil O. Kapoor R. Davies K. J. Lowry C. V. (2001). Regulatory mechanisms controlling expression of the DAN/TIR mannoprotein genes during anaerobic remodeling of the cell wall in *Saccharomyces cerevisiae*. *Genetics.* 157 1169–1177. 11238402 10.1093/genetics/157.3.1169 PMC1461566

[B3] Albergaria H. Francisco D. Gori K. Arneborg N. Gírio F. (2010). *Saccharomyces cerevisiae* CCMI 885 secretes peptides that inhibit the growth of some non-*Saccharomyces* wine-related strains. *Appl. Microbiol. Biotechnol.* 86 965–972. 10.1007/s00253-009-2409-6 20039034

[B4] Albertin W. Setati M. E. Miot-Sertier C. Mostert T. T. Colonna-Ceccaldi B. Coulon J. (2015). *Hanseniaspora uvarum* from winemaking environments show spatial and temporal genetic clustering. *Front. Microbiol.* 6:1569 10.3389/fmicb.2015.01569 PMC4718985 26834719

[B5] Anders S. Py P. T. Huber W. (2015). HTSeq–a Python framework to work with high-throughput sequencing data. *Bioinformatics* 31 166–169. 10.1093/bioinformatics/btu638 25260700 PMC4287950

[B6] Balakrishnan R. Park J. Karra K. Hitz B. C. Binkley G. Hong E. L. (2012). YeastMine–an integrated data warehouse for *Saccharomyces cerevisiae* data as a multipurpose tool-kit. *Database* 2012:bar062. 10.1093/database/bar062 22434830 PMC3308152

[B7] Barbosa C. Mendes-Faia A. Lage P. Mira N. P. Mendes-Ferreira A. (2015). Genomic expression program of *Saccharomyces cerevisiae* along a mixed-culture wine fermentation with *Hanseniaspora guilliermondii*. *Microb. Cell Fact.* 14:124. 10.1186/s12934-015-0318-1 26314747 PMC4552253

[B8] Belda I. Navascués E. Marquina D. Santos A. Calderon F. Benito S. (2015). Dynamic analysis of physiological properties of Torulaspora delbrueckii in wine fermentations and its incidence on wine quality. *Appl. Microbiol. Biotechnol.* 99 1911–1922. 10.1007/s00253-014-6197-2 25408314

[B9] Branco P. Francisco D. Chambon C. Hébraud M. Arneborg N. Almeida M. G. (2014). Identification of novel GAPDH-derived antimicrobial peptides secreted by *Saccharomyces cerevisiae* and involved in wine microbial interactions. *Appl. Microbiol. Biotechnol.* 98 843–853. 10.1007/s00253-013-5411-y 24292082

[B10] Ciani M. Capece A. Comitini F. Canonico L. Siesto G. Romano P. (2016). Yeast interactions in inoculated wine fermentation. *Front. Microbiol.* 7:555 10.3389/fmicb.2016.00555 PMC4840204 27148235

[B11] Ciani M. Comitini F. (2011). Non-*Saccharomyces* wine yeasts have a promising role in biotechnological approaches to winemaking. *Ann. Microbiol.* 61 25–32. 10.1007/s13213-010-0069-5

[B12] Emig D. Salomonis N. Baumbach J. Lengauer T. Conklin B. R. Albrecht M. (2010). AltAnalyze and DomainGraph: analyzing and visualizing exon expression data. *Nucleic Acids Res.* 38 W755–W762. 10.1093/nar/gkq405 20513647 PMC2896198

[B13] Fleet G. H. (2003). Yeast interactions and wine flavour. *Int. J. Food Microbiol.* 86 11–22. 10.1016/S0168-1605(03)00245-9 12892919

[B14] Gancedo J. M. (1998). Yeast carbon catabolite repression. *Microbiol. Mol. Biol. Rev.* 62 334–361. 9618445 10.1128/mmbr.62.2.334-361.1998 PMC98918

[B15] Jolly N. P. Varela C. Pretorius I. S. (2014). Not your ordinary yeast: non-Saccharomyces yeasts in wine production uncovered. *FEMS Yeast Res.* 14 215–237. 10.1111/1567-1364.12111 24164726

[B16] Kayikci Ö. Nielsen J. (2015). Glucose repression in *Saccharomyces cerevisiae*. *FEMS Yeast Res.* 15:fov068. 10.1093/femsyr/fov068 26205245 PMC4629793

[B17] Kim D. Pertea G. Trapnell C. Pimentel H. Kelley R. Salzberg S. L. (2013). TopHat2: accurate alignment of transcriptomes in the presence of insertions, deletions and gene fusions. *Genome Biol.* 14:R36. 10.1186/gb-2013-14-4-r36 23618408 PMC4053844

[B18] Magasanik B. Kaiser C. A. (2002). Nitrogen regulation in *Saccharomyces cerevisiae*. *Gene* 290 1–18. 10.1016/S0378-1119(02)00558-9 12062797

[B19] Masneuf-Pomarede I. Bely M. Marullo P. Albertin W. (2016). The genetics of non-conventional wine yeasts: current knowledge and future challenges. *Front. Microbiol.* 6:1563. 10.3389/fmicb.2015.01563 26793188 PMC4707289

[B20] Maturano Y. P. Assof M. Fabani M. P. Nally M. C. Jofré V. Assaf L. A. R. (2015). Enzymatic activities produced by mixed Saccharomyces and non-*Saccharomyces* cultures: relationship with wine volatile composition. *Antonie Van Leeuwenhoek* 108 1239–1256. 10.1007/s10482-015-0578-0 26386703

[B21] Morales P. Rojas V. Quirós M. González R. (2015). The impact of oxygen on the final alcohol content of wine fermented by a mixed starter culture. *Appl. Microbiol. Biotechnol.* 99 3993–4003. 10.1007/s00253-014-6321-3 25582558 PMC4428804

[B22] Oliveros J. C. (2007–2015). *Venny. An interactive Tool for Comparing Lists with Venn’s Diagrams*. Available at: http://bioinfogp.cnb.csic.es/tools/venny/index.html

[B23] Ozcan S. (2002). Two different signals regulate repression and induction of gene expression by glucose. *J. Biol. Chem.* 277 46993–46997. 10.1074/jbc.M208726200 12351652

[B24] Pérez F. Ramírez M. Regodón J. A. (2001). Influence of killer strains of *Saccharomyces cerevisiae* on wine fermentation. *Antonie van Leeuwenhoek* 79 393–399. 10.1023/A:1012034608908 11816985

[B25] Pérez-Torrado R. Rantsiou K. Perrone B. Navarro-Tapia E. Querol A. Cocolin L. (2017). Ecological interactions among *Saccharomyces cerevisiae* strains: insight into the dominance phenomenon. *Sci. Rep.* 7:43603. 10.1038/srep43603 28266552 PMC5339867

[B26] Renault P. Coulon J. de Revel G. Barbe J. C. Bely M. (2015). Increase of fruity aroma during mixed T. delbrueckii/S. cerevisiae wine fermentation is linked to specific esters enhancement. *Int. J. Food Microbiol.* 207 40–48. 10.1016/j.ijfoodmicro.2015.04.037 26001522

[B27] Renault P. Coulon J. Moine V. Thibon C. Bely M. (2016). Enhanced 3-sulfanylhexan-1-ol production in sequential mixed fermentation with *Torulaspora delbrueckii*/*Saccharomyces cerevisiae* reveals a situation of synergistic interaction between two industrial strains. *Front. Microbiol.* 7:293. 10.3389/fmicb.2016.00293 27014216 PMC4792154

[B28] Rivero D. Berná L. Stefanini I. Baruffini E. Bergerat A. Csikász-Nagy A. (2015). Hsp12p and PAU genes are involved in ecological interactions between natural yeast strains. *Environ. Microbiol.* 17 3069–3081. 10.1111/1462-2920.12950 26079802

[B29] Robinson M. D. McCarthy D. J. Smyth G. K. (2010). edgeR: a Bioconductor package for differential expression analysis of digital gene expression data. *Bioinformatics* 26 139–140. 10.1093/bioinformatics/btp616 19910308 PMC2796818

[B30] Robinson M. D. Oshlack A. (2010). A scaling normalization method for differential expression analysis of RNA-seq data. *Genome Biol.* 11:R25. 10.1186/gb-2010-11-3-r25 20196867 PMC2864565

[B31] Rodrigues A. J. Raimbaud T. Gonzalez R. Morales P. (2016). Environmental factors influencing the efficacy of different yeast strains for alcohol level reduction in wine by respiration. *LWT Food Sci. Technol.* 65 1038–1043. 10.1016/j.lwt.2015.09.046

[B32] Rossouw D. Bagheri B. Setati M. E. Bauer F. F. (2015). Co-flocculation of yeast species, a new mechanism to govern population dynamics in microbial ecosystems. *PLOS ONE* 10:e0136249. 10.1371/journal.pone.0136249 26317200 PMC4552943

[B33] Rossouw D. Toit Du M. Bauer F. F. (2012). The impact of co-inoculation with *Oenococcus oeni* on the transcriptome of *Saccharomyces cerevisiae* and on the flavour-active metabolite profiles during fermentation in synthetic must. *Food Microbiol.* 29 121–131. 10.1016/j.fm.2011.09.006 22029926

[B34] Sertil O. Cohen B. D. Davies K. J. Lowry C. V. (1997). The DAN1 gene of S. cerevisiae is regulated in parallel with the hypoxic genes, but by a different mechanism. *Gene* 19 199–205. 10.1016/S0378-1119(97)00028-0 9224891

[B35] Tai S. L. Boer V. M. Daran-Lapujade P. Walsh M. C. de Winde J. H. Daran J. M. (2005). Two-dimensional transcriptome analysis in chemostat cultures combinatorial effects of oxygen availability and macronutrient limitation in *Saccharomyces cerevisiae*. *J. Biol. Chem.* 7 437–447. 10.1074/jbc.M410573200 15496405

[B36] Taillandier P. Lai Q. P. Julien-Ortiz A. Brandam C. (2014). Interactions between *Torulaspora delbrueckii* and *Saccharomyces cerevisiae* in wine fermentation: influence of inoculation and nitrogen content. *World J. Microb. Biot.* 30 1959–1967. 10.1007/s11274-014-1618-z 24500666

[B37] ter Linde J. J. Liang H. Davis R. W. Steensma H. Y. van Dijken J. P. Pronk J. T. (1999). Genome-wide transcriptional analysis of aerobic and anaerobic chemostat cultures of *Saccharomyces cerevisiae*. *J. Bacteriol.* 181 7409–7413. 10601195 10.1128/jb.181.24.7409-7413.1999 PMC94195

[B38] Trapnell C. Hendrickson D. G. Sauvageau M. Goff L. Rinn J. L. Pachter L. (2013). Differential analysis of gene regulation at transcript resolution with RNA-seq. *Nat. Biotechnol.* 31 46–53. 10.1038/nbt.2450 23222703 PMC3869392

[B39] Tristezza M. Tufariello M. Capozzi V. Spano G. Mita G. Grieco F. (2016). The oenological potential of *Hanseniaspora uvarum* in simultaneous and sequential co-fermentation with *Saccharomyces cerevisiae* for industrial wine production. *Front. Microbiol.* 7:670. 10.3389/fmicb.2016.00670 27242698 PMC4860541

[B40] Tronchoni J. Curiel J. A. Morales P. Torres-Pérez R. Gonzalez R. (2017). Early transcriptional response to biotic stress in mixed starter fermentations involving *Saccharomyces cerevisiae* and *Torulaspora delbrueckii*. *Int. J. Food Microbiol.* 241 60–68. 10.1016/j.ijfoodmicro.2016.10.017 27756034

[B41] van Vuuren H. Jacobs C. J. (1992). Killer yeasts in the wine industry: a review. *Am. J. Enol. Vitic.* 43 119–128.

[B42] Velázquez R. Zamora E. Álvarez M. L. Hernández L. M. Ramírez M. (2015). Effects of new *Torulaspora delbrueckii* killer yeasts on the must fermentation kinetics and aroma compounds of white table wine. *Front. Microbiol.* 6:1222. 10.3389/fmicb.2015.01222 26579114 PMC4630308

[B43] Wang C. Mas A. Esteve-Zarzoso B. (2015). Interaction between *Hanseniaspora uvarum* and *Saccharomyces cerevisiae* during alcoholic fermentation. *Int. J. Food Microbiol.* 206 67–74. 10.1016/j.ijfoodmicro.2015.04.022 25956738

[B44] Wang C. Mas A. Esteve-Zarzoso B. (2016). The interaction between *Saccharomyces cerevisiae* and non-*Saccharomyces* yeast during alcoholic fermentation is species and strain specific. *Front. Microbiol.* 7:502. 10.3389/fmicb.2016.00502 27148191 PMC4829597

[B45] Yang X. Li J. Lee Y. Lussier Y. A. (2011). GO-module: functional synthesis and improved interpretation of gene ontology patterns. *Bioinformatics* 27 1444–1446. 10.1093/bioinformatics/btr142 21421553 PMC3087953

